# Krüppel-like factor 4 modulates the miR-101/COL10A1 axis to inhibit renal fibrosis after AKI by regulating epithelial–mesenchymal transition

**DOI:** 10.1080/0886022X.2024.2316259

**Published:** 2024-02-12

**Authors:** Jingying Zhao, Xiuli Wang, Yubin Wu, Chengguang Zhao

**Affiliations:** Department of Pediatrics, Shengjing Hospital of China Medical University, Shenyang, People’s Republic of China

**Keywords:** COL10A1, renal fibrosis, EMT, KLF4, miR-101

## Abstract

Acute kidney injury (AKI) can progress to renal fibrosis and chronic kidney disease (CKD), which reduces quality of life and increases the economic burden on patients. However, the molecular mechanisms underlying renal fibrosis following AKI remain unclear. This study tested the hypothesis that the Krüppel-like factor 4 (KLF4)/miR-101/Collagen alpha-1X (COL10A1) axis could inhibit epithelial-mesenchymal transition (EMT) and renal fibrosis after AKI in a mouse model of ischemia-reperfusion (I/R)-induced renal fibrosis and HK-2 cells by gene silencing, overexpression, immunofluorescence, immunohistochemistry, real-time quantitative PCR, Western blotting, dual-luciferase reporter assay, fluorescence *in situ* hybridization (FISH) and ELISA. Compared with the Sham group, I/R induced renal tubular and glomerular injury and fibrosis, and increased the levels of BUN, serum Scr and neutrophil gelatinase-associated lipocalin (NGAL), Col10a1 and Vimentin expression, but decreased E-cadherin expression in the kidney tissues of mice at 42 days post-I/R. Similarly, hypoxia promoted fibroblastic morphological changes in HK-2 cells and enhanced NGAL, COL10A1, Vimentin, and α-SMA expression, but reduced E-cadherin expression in HK-2 cells. These pathological changes were significantly mitigated in COL10A1-silenced renal tissues and HK-2 cells. KLF4 induces miR-101 transcription. More importantly, hypoxia upregulated Vimentin and COL10A1 expression, but decreased miR-101, KLF4, and E-cadherin expression in HK-2 cells. These hypoxic effects were significantly mitigated or abrogated by KLF4 over-expression in the HK-2 cells. Our data indicate that KLF4 up-regulates miR-101 expression, leading to the downregulation of COL10A1 expression, inhibition of EMT and renal fibrosis during the pathogenic process of I/R-related renal fibrosis.

## Introduction

The prevalence of acute kidney injury (AKI) and chronic kidney disease (CKD) is increasing worldwide. AKI can progress to CKD in the absence of optimal treatments [1]. Previous studies have shown that AKI can impair kidney function that may be incompletely recovered, chronically progressing into tubulointerstitial renal fibrosis, which is a unique pathological characteristic of AKI to CKD. However, the mechanism by which AKI progresses to renal fibrosis is not fully understood [2,3]. Induction of hypoxia is a classical method for inducing renal fibrosis in rodents [4–6]. Ischemia/reperfusion (I/R) can induce AKI and damage tubular epithelial cells in the kidney. Although tubular epithelial cells in the kidney can be regenerated and functionally repaired for the recovery of renal structure and function, they can undergo epithelial–mesenchymal transition (EMT), affecting their recovery and leading to renal fibrosis [7,8]. Hence, understanding the mechanisms underlying the EMT process is of significance in preventing renal fibrosis after AKI.

Renal tubular epithelial cells undergo EMT process and transdifferentiate into myofibroblasts under specific conditions [9], which are crucial for renal fibrosis. Some potential drugs have been evaluated in mouse models of AKI: the DPP-4 inhibitor teneligliptin accelerates recovery from the cisplatin-induced AKI by promoting the proliferation of renal proximal tubular epithelial cells (RPTEC), and CXCL12, one of the DPP-4 substrate chemokines, is a responsible mitogen of RPTEC proliferation [4]. SIRT3 protects against tubular injury in an animal model of AKI by regulating mitochondrial dynamics [5]. Treatment with glycolytic inhibitors to inhibit lactate production in tubules suppresses fibroblast activation following folic acid-induced renal injury [6]. In rodents, mineralocorticoid receptor antagonism prevents the I/R-induced AKI [10]. Our previous study has shown that upregulated miR-101 expression inhibits the EMT process of renal epithelial cells and the expression of collagen type X alpha 1 (COL10A1), which is one of the targets of miR-101 [11]. COL10A1 is highly expressed in various cancers. Upregulated COL10A1 expression is associated with the invasion, migration, and EMT processes of cancer cells [12,13]. However, there is no information on whether COL10A1 can modulate the process of EMT in renal epithelial cells during the process of renal fibrosis after AKI. Kruppel-like factor 4 (KLF4), a transcription factor, is crucial for renal fibrosis and kidney injury, and its expression is downregulated during kidney injury [14]. It is predicted that KLF4 can bind to the promoter region of pre-miR-101. Accordingly, we speculated that KLF4 might regulate the expression of miR-101. Therefore, we tested whether KLF4 could modulate the miR-101/COL10A1 axis to affect renal fibrosis after AKI in mice and HK-2 cells.

## Materials and methods

### Animals

Male C57BL/6 mice (6–8 weeks old) were obtained from Liaoning Changsheng Biotechnology (Benxi, China). The mice were divided into four groups: Sham, I/R, I/R + negative control (si-NC), and I/R + si-Col10a1 (si-Col10a1, *n* = 10 mice per group). The renal I/R injury model was generated according to a previous report [15]. Briefly, the mice were anesthetized, and the left renal hilum of individual mice was exposed without clamping as in the sham group or clamped for 35 min and released to induce I/R injury. One day later, the I/R mice were further randomized and injected intravenously, with or without (I/R group) 1.5 × 10^8^ TU lentivirus (150 µL/mouse) for the expression of control scramble siRNA (siNC group) or *Col10a1*-specific siRNA (si-Col10a1 group, GenePharma Shanghai, China). Five weeks later, contralateral kidneys were surgically removed. Six weeks later, the mice were euthanized and peripheral blood samples were collected. Subsequently, their kidneys were dissected. The sequences of control and *Col10a1*-sepcific siRNAs were sense UUCUCCGAACGUGUCACGUTT and antisense ACGUGACACGUUCGGAGAATT, and sense CAUCAAAGGUGAUAGAGGUTT and antisense ACCUCUAUCACCUUUGAUGTT, respectively.

### Cell culture

Human kidney proximal tubular HK-2 cells were obtained from the Chinese Academy of Sciences and maintained in DMEM supplemented with 10% fetal bovine serum (FBS). HK-2 cells were cultured in a hypoxic incubator of 1% O_2_, 5% CO_2_, 94% N2 at 37 °C for 48 h to generate a hypoxic cellular model, and other HK-2 cells were cultured in a normoxic incubator of 21% O_2_, 5% CO_2_ at 37 °C for 48h to form a normoxic cellular model

### Luciferase assay

293T cells were transfected with the plasmid pmirGLO-miR-101 and the luciferase reporter containing either the wild-type or mutant 3′-UTR sequence of *COL10A1* using Lipofectamine 2000 (11668-019, Invitrogen). In addition, 293 T cells were transfected with pGL3-Basic/KLF4 together with the plasmid for the wild-type pre-miR-101 promoter or its mutant-controlled luciferase reporter. Two days later, their luciferase activity was measured using a Dual-Luciferase Reporter Assay Kit, according to the manufacturer’s instruction (KGAF040, Netherlands).

### Blood urea nitrogen and serum creatinine measurements

Blood samples were prepared for their serum samples. Blood urea nitrogen (BUN) and serum creatinine (Scr) levels in individual mice were determined using an automatic biochemical analyzer (model ci16200, Abbott Laboratories).

### HE and Masson trichrome staining

Kidney tissues from individual mice were fixed with 4% paraformaldehyde and embedded in paraffin. Kidney tissue sections (3 μm) were routinely stained with hematoxylin and eosin (H&E), or Masson’s trichrome solution. Renal histological changes, the renal tubular injury index, and fibrotic areas were observed under a light microscope.

### Enzyme-linked immunosorbent assay (ELISA)

The concentrations of serum neutrophil gelatinase-associated lipocalin (NGAL) in individual mice were measured by ELISA using a specific kit (Uscnk, China) following the supplier’s protocol.

Immunohistochemistry

The kidney tissue Sections (3 µm) were dewaxed, rehydrated, and subjected to the antigen retrieval. The sections were incubated with anti-COL10A1 and anti-KLF4 antibody (1:1000, Novus Biologicals, USA) at 4 °C overnight. The bound antibodies were reacted with horseradish peroxidase (HRP)-labeled goat anti-rabbit IgG and visualized with DAB (3,3′-Diaminobenzidine), followed by counterstained with hematoxylin.

### Fluorescence *in situ* hybridization (FISH)

The distribution of miR-101 expression was identified by FISH. Briefly, the paraffin-embedded kidney tissue sections (5 µm) were dewaxed, rehydrated and digested with protease K, followed by dehydrated. The sections were heat-denatured and prehybridized with buffer E. Subsequently, the sections were hybridized with biotinylated miR-101 anti-sense probe (Genepharma, Shanghai, China) and Cy3-labeled streptavidin for at 43 °C for 12 h. After being washed, the sections were nuclearly stained with DAPI and the hybridization signals were observed under a fluorescent microscope.

### Immunofluorescence (IF) staining

HK-2 cells were cultured on glass slips and fixed with 4% paraformaldehyde for 20 min. The cells were permeabilized with 0.1% Triton X-100 for 20 min and treated with 3% goat serum albumin in PBS. The HK-2 cells were probed with anti-E-cadherin or anti-vimentin antibodies (1:100, CST) as well as the vehicle control PBS at 4 °C overnight. The bound antibodies were reacted with fluorescent-secondary antibodies and the cells were nuclear-stained with 0.1% DAPI. Similarly, the levels of E-cadherin and Vimentin expression in the kidney tissues were also examined by immunofluorescence. Fluorescent signals were captured and evaluated under a fluorescent microscope (Nikon).

### Cell transfection

HK-2 cells were transfected with si-COL10A1 or control scramble siRNA (JTS Scientific Biotechnology, China) using Lipofectamine 2000 [11]. The sequences of siRNAs were CAUCAAAGGUGAUAGAGGUTT for COL10A1-specific siRNA and UUCUCCGAACGUGUCACGUTT for control scramble siRNA. HK2 cells were transfected with either the pcDNA3.1-KLF4 construct or the control pcDNA3.1. Two days later, the cells were harvested and tested.

### Western blotting

Fresh kidney tissues were homogenized and HK-2 cells were lysed in lysis buffer containing proteinase inhibitors. After centrifugation, the protein concentrations of each homogenate or cell lysate were measured. Individual samples (30 µg/lane) were separated by sodium dodecyl sulfate-polyacrylamide gel electrophoresis on 10% gels and electronically transferred onto polyvinylidene difluoride membranes (Millipore, Billerica, MA, USA). After being treated with 5% fat-free milk in TBST, the membranes were probed with primary antibodies overnight at 4 °C, including anti-α-SMA (1:500, Proteintech, China), anti-E-cadherin (1:500, CST), anti-vimentin (1:500, CST), anti-COL10A1 (1:500, ABclonal), anti-NGAL (1:500, Proteintech, China), anti-KLF4 (1:500, CST), anti-COLI (1:500, ABclonal) and anti-GAPDH (1:10000, Proteintech). Subsequently, the membranes were incubated with goat anti-rabbit antibodies (1:3000, Solarbio) and visualized using enhanced chemiluminescence (ECL, Amersham, UK). The data were analyzed by densitometric scanning using the ImageJ software.

### RT-qPCR

We extracted all RNAs from kidney tissues and HK-2 cells using TRIzol reagent (Takara, Shiga, Japan) and a total RNA isolation kit (Takara, Japan). RNA samples were reverse-transcribed into complementary DNA using an RT Primer (Takara, Japan). The relative levels of target gene to the control GAPDH mRNA transcripts were quantified in triplicate by RT-qPCR using specific primers. The generated data were analyzed using the 2^-△△Ct^ method.

### Statistical analysis

All data are presented as mean ± standard deviation (SD). The data were analyzed by one-way analysis of variance and two-tailed unpaired Student’s *t*-test, where applicable, using SPSS 21.0. Statistical significance was set at a *p*-value of <0.05.

## Results

### Down-regulated miR-101, E-cadherin and KLF4 expression is associated with up-regulated Vimentin and COL10A1 expression in I/R mouse kidney tissues

**Figure 1.**
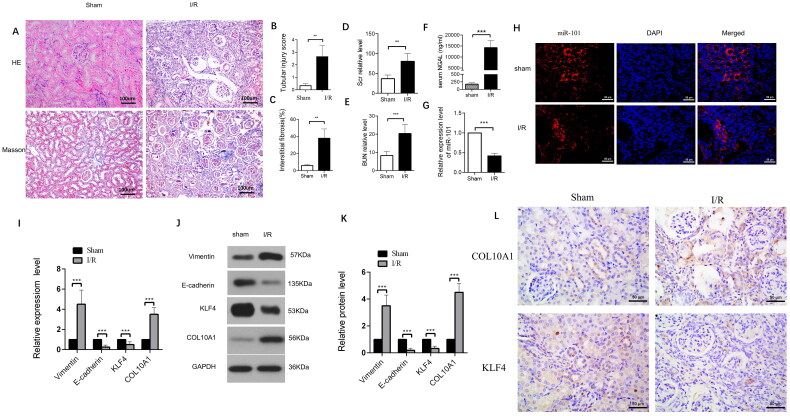
The I/R induces the EMT process and renal fibrosis in mice. (A) The representative images of kidney sections from each group of mice after H&E and Masson staining (magnification x 400); (B–C) Renal tubular injury index and fibrotic areas; (D–E) Scr and BUN levels in the indicated groups of mice; (F) NGAL levels in the indicated groups of mice; (G) The levels of miR-101 transcripts in mice. (H) FISH analysis of miR-101 transcripts in kidney tissue sections. (I) RT-qPCR analysis of the relative levels of Vimentin, E-cadherin, COL10A1 and KLF4 mRNA transcripts in kidney tissues of the Sham and I/R groups of mice; (J, K) Western blot analysis of the relative levels of Vimentin, E-cadherin, COL10A1 and KLF4 protein expression in kidney tissues of the Sham and I/R groups of mice. (L) Immunohistochemistry analysis of COL10A1 and KLF4 expression in kidney tissues of both groups of mice. Data are presented as means ± SD of each group (n = 10). *p < 0.05, **p < 0.01, ***p < 0.001.

To understand the pathogenesis of renal fibrosis after AKI, we generated a mouse model of I/R-induced renal injury, and 42 days later, we characterized the changes in renal tissues. The gross kidney organs differed between the I/R and Sham groups. In the I/R group, the kidney organs were smaller, with reduced weights and thinning of the cortical layer, compared with the sham group (Figure 1A). Histological examination revealed substantial accumulation of inflammatory infiltrates and obvious vascular cell proliferation in the kidney tissues of the I/R groups of mice, accompanied by damaged glomerular structure, compared to the sham controls. Quantitative analysis indicated that the renal tubular injury scores and the percentages of fibrotic areas were significantly greater in the I/R groups than in the sham group (Figure 1B–C). Measurements of serum Scr and BUN levels indicated that the levels of BUN and serum Scr in the I/R group were significantly higher than those in the sham group 7 days after removal of the contralateral kidney (*p* < 0.01, *p* < 0.001, Figure 1D–E). In addition, the levels of serum NGAL in the I/R group were significantly higher than those in the Sham group at 42 days post I/R injury (*p* < 0.001, Figure 1F). In contrast, the levels of miR-101 transcripts in the kidney tissues from the I/R group were lower than those in the sham group (Figure 1G). FISH revealed that miR-101 was mainly expressed in the cytoplasm of renal tubular cells, and the relative levels of miR-101 signals in the I/R group were obviously lower than that in the Sham group of mice (Figure 1H). Western blotting and RT-qPCR analyses revealed that, compared with the Sham group of mice, the relative levels of Col10a1 and Vimentin mRNA transcripts and protein expression increased, while E-cadherin and KLF4 decreased in kidney tissues from the I/R group of mice (Figure 1I–K). Immunohistochemistry displayed that compared with the Sham group, up-regulated COL10A1 expression was mainly detected in the cytoplasm and down-regulated KLF4 expression was centered on the nucleus of renal tubular cells of mice (Figure 1L). Collectively, I/R-induced AKI promoted renal fibrosis in mice at 42 days post-induction by enhancing the EMT process in the kidney of mice and down-regulating the expression of KLF4 and miR-101 to enhance COL10A1 expression.

### COL10A1 is the target of miR-101

**Figure 2.**
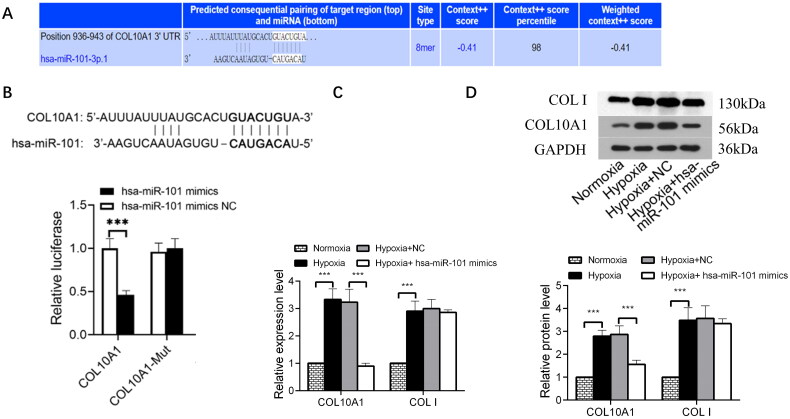
COL10A1 is one of the targets of miR-101. (A) COL10A1 was predicted as one of the potential targets of miR-101 in TargetScan (http://www.targetscan.org). (B) Conserved miR-101-binding sites in the 3’-UTRs of COL10A1 mRNA. Luciferase reporter assays reported that transfection of miR-101 mimic inhibited the wild-type COL10A1 3’UTR, but not its mutant-regulated luciferase expression in the 293T cells. (C) Effects of miR-101 mimics on the levels of endogenous COL10A1 and COLI mRNA transcripts in HK-2 cells under the indicated conditions, determined by RT-qPCR. (D) Transfection with miR-101 mimics decreased the levels of endogenous COL10A1 protein expression in HK-2 cells under the indicated conditions, but not COL I. Data are presented as means ± SD of each group from three separate experiments. *p < 0.05, **p < 0.01, ***p < 0.001; n =3.

We predicted COL10A1 as a potential target of miR-101 using TargetScan (http://www.targetscan.org) (Figure 2A). After constructing luciferase reporter plasmids carrying the wild-type or mutant COL10A1 3′-UTR sequence for miR-101 binding, we examined the regulatory role of miR-101 in COL10A1 3′-UTR-regulated luciferase expression in 293 T cells using a dual-luciferase assay. The results indicated that miR-101 overexpression inhibited the luciferase activity in the cells that had been transfected with the plasmid for the expression of wild-type COL10A1 3′-UTR, but not the plasmid of mutant COL10A1 3′-UTR (Figure 2B). Similarly, transfection with an hsa-miR-101 mimic reduced the levels of COL10A1, but not collagen I, mRNA transcripts and protein expression in HK-2 cells under a hypoxic condition (Figure 2C and D). These results suggest that *COL10A1* may be a target of miR-101.

### Col10a1 is crucial for the I/R-induced renal injury and fibrosis in mice by enhancing the EMT process

**Figure 3.**
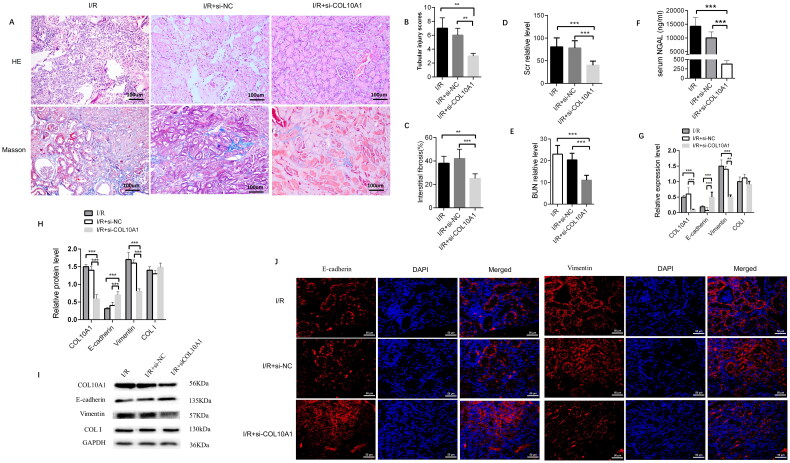
Col10a1 Is crucial for the I/R-induced renal injury, fibrosis and EMT process in the AKI-CKD mice. (A) Representative images of HE and Masson’s trichrome-stained kidney sections (bars = 100 μm). (B) Renal tubular injury scores. (C) Fibrotic areas. (D-E) The levels of BUN and Scr. (F) ELISA measured the levels of serum NGAL in mice. (G) the relative levels of Col10a1, ColI, E-cadherin and Vimentin mRNA transcripts in kidney tissues. (H-I) Western blotting analysis of the relative levels of Col10a1, ColI, E-cadherin and Vimentin expression in the kidney tissues of mice. (J) Immunofluorescence analysis of E-cadherin and Vimentin expression in kidney tissue sections from the indicated groups of mice. Data are representative images or presented as means ± SD of each group (n = 10) from three independent experiments. ** *p* < 0.01, *** *p* < 0.001.

I/R mice were randomized and intravenously injected with the control lentivirus (I/R + si-NC group) or lentivirus-mediated *ColA10a1* silencing (I/R + si-COL10A1 group). Six weeks later, we characterized histopathological changes in the kidney tissues of the different groups of mice. Masson’s trichrome staining revealed that COL10A1 silencing attenuated glomerulosclerosis and interstitial fibrosis. There was a large area of collagen deposition and extracellular matrix accumulation in the kidney sections of the I/R and I/R + si-NC groups, indicating that I/R induced glomerulosclerosis and interstitial fibrosis. In contrast, the degrees of glomerulosclerosis and interstitial fibrosis were attenuated in the I/R + si-Col10a1 group (Figure 3A). Quantitative analysis indicated that the renal tubular injury scores and the percentages of fibrotic areas were significantly greater in the I/R and I/R + si-NC groups than in the I/R + si-Col10a1 group (Figure 3B–C). Hence, *Col10a1* silencing mitigated the I/R-induced renal injury and fibrosis in the mice. The levels of BUN and serum Scr in the I/R + si-Col10a1 group were significantly lower than those in the I/R and I/R + si-NC groups of mice after removal of contralateral kidneys seven days before measurements (*p* < 0.001, Figure 3D–E). Furthermore, serum NGAL levels were lower in the I/R + si-Col10a1 group than in the I/R and I/R + si-NC groups (*p* < 0.001, Figure 3F). Next, we measured the relative levels of E-cadherin, Vimentin and collagen I expression in kidney tissues to explore the impact of *Col10a1* silencing on EMT in mice. Compared with the I/R and I/R-si-NC groups of mice, the relative levels of Col10a1 and Vimentin mRNA transcripts and protein expression decreased, E-cadherin increased while Collagen I expression did not change in the kidney tissues of the I/R-si-Col10a1 group of mice (Figure 3G–I). A similar pattern of Col10a1, Vimentin and E-cadherin expression was detected in the kidney tissues of different groups of mice by immunofluorescence (Figure 3J). Collectively, Col10a1 silencing mitigated renal fibrosis in I/R mice by inhibiting AKI-enhanced EMT in the kidneys.

### COL10A1 silencing mitigates the hypoxia-up-regulated NGAL expression in HK-2 cells

**Figure 4.**
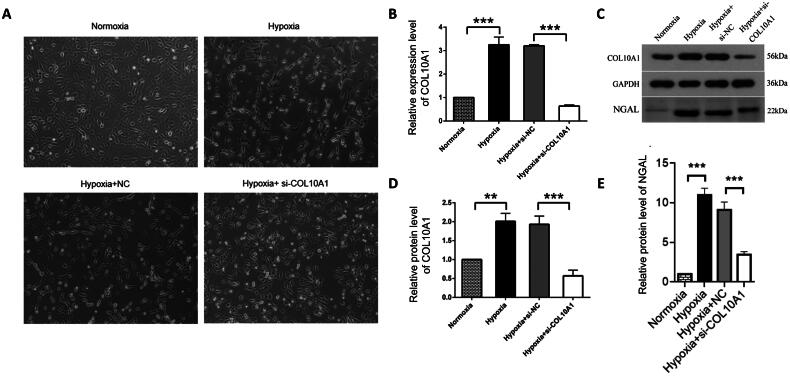
COL10A1 silencing mitigates the hypoxia-induced morphological changes and NGAL expression in HK-2 cells. HK-2 cells were transfected with control scrambled siRNA (NC) or COL10A1-specific siRNA for 48 h. Different groups of cells were cultured under a normoxic or hypoxic condition for 48 h. The cell morphology and relative levels of COL10A1 and NGAL expression in different groups of cells were examined. (A) Morphological observations of different groups of cells (magnification x100). (B) RT-qPCR analysis of COL10A1 mRNA transcripts in the different cell groups. (C–E) Western blot analysis of COL10A1 and NGAL expression in different groups of HK-2 cells. Data are representative images or are presented as the mean ± SD of each group (n = 3) from three independent experiments, ** *p* < 0.01, *** *p* < 0.001.

HK-2 and HK-2-NC cells under a hypoxic condition for 48 h resulted in morphological changes from an oval paving stone shape to a long spindle fibroblast-like shape, whereas COL10A1-silenced HK-2 cells in the hypoxic condition retained partial epithelioid morphology (Figure 4A). In addition, hypoxia increased the relative levels of *COL10A1* mRNA transcripts and protein expression in HK-2 cells, which were dramatically reduced in *COL10A1*-silenced HK-2 cells (Figure 4B–D). More importantly, hypoxia significantly increased the levels of NGAL secreted by cultured cells, which were also significantly reduced in the supernatants of cultured *COL10A1*-silenced HK-2 cells (Figure 4E). Hence, *COL10A1* silencing significantly mitigated the hypoxia-upregulated NGAL expression in HK-2 cells.

### COL10A1 silencing inhibits the EMT process in hypoxic HK-2 cells

**Figure 5.**
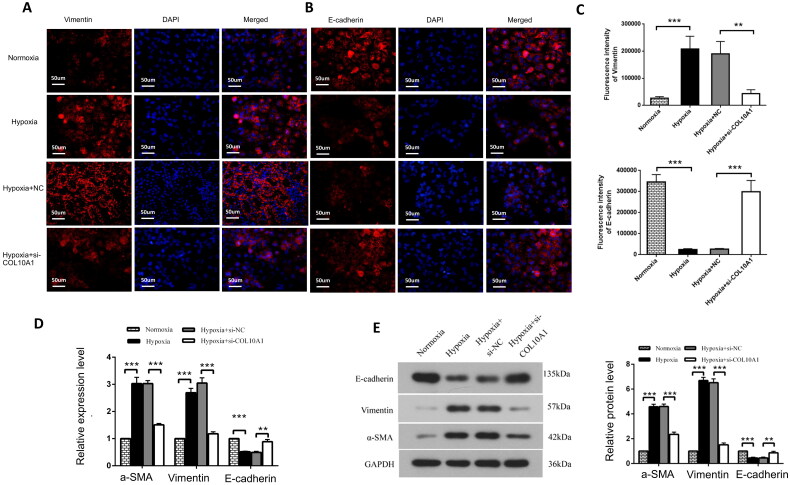
COL10A1 silencing inhibits the EMT process induced by hypoxia in HK-2 cells. (A, B) Immunofluorescent analysis of E-cadherin and Vimentin expression in different groups of cells. Bar = 50 μm. (C) Quantitative analysis of fluorescent signals. (D–E) Quantitative analysis of E-cadherin, Vimentin and αSMA expression in the indicated groups of cells. Data are representative images or presented as means ± SD of each group from three independent experiments. * *p* < 0.05, ** *p* < 0.01, *** *p* < 0.001.

hypoxia decreased E-cadherin expression, but increased vimentin expression in HK-2 cells, which was significantly mitigated or abrogated in *COL10A1*-silenced HK-2 cells (Figure 5A–C). Similar patterns of Vimentin, E-cadherin and α-SMA expression were observed in different groups of cells by Western blotting and RT-qPCR and *COL10A1* silencing significantly mitigated the hypoxia-induced increase in α-SMA expression in HK-2 cells (Figure 5D and E). Therefore, COL10A1 silencing attenuated the hypoxia-induced EMT in HK-2 cells.

### KLF4 up-regulates miR-101 expression to inhibit COL10A1 expression and EMT process in HK-2 cells

**Figure 6.**
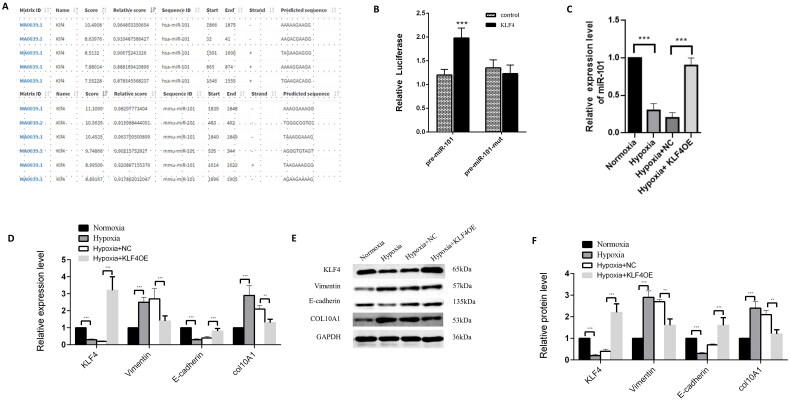
KLF4 induces miR-101 expression to minimize COL10A1 expression and the EMT process in HK-2 cells. JASPAR website predicted the binding KLF4 to the miR-101 promoter region. (B) Dual-luciferase reporter assays indicated that KLF4 induced pre-miR-101 transcription in 293T cells. The pre-miR-101 indicates the pre-miR-101 promoter. The pre-miR-101 mut represents the mutant pre-miR-101 promoter. (C) KLF4 overexpression increased miR-101 expression in HK-2 cells. (D–F) RT-qPCR and Western blotting analyses of the relative levels of COL10A1 expression and EMT in the indicated groups of HK-2 cells. Data are representative images or presented as the mean ± SD of each group from three independent experiments. ** *p* < 0.01, *** *p* < 0.001.

KLF4 can bind to the promoter of premiR-101, based on our preliminary bioinformatics study (Figure 6A). Transfection with the plasmid for KLF4 over-expression, but not the control plasmid, together with the plasmid for the wild-type pre-miR-101 promoter-controlled luciferase, increased luciferase activity by 65% in 293 T cells, whereas induction of KLF4 over-expression did not affect the mutant pre-miR-101 promoter-controlled luciferase expression in 293 T cells (Figure 6B). These data clearly indicate that KLF4 is a major transcription factor that induces pre-miR-101 expression. Furthermore, we tested the impact of KLF4 overexpression on miR-101 and COL10A1 expression, as well as the EMT process in HK-2 cells in a hypoxic condition, by RT-qPCR and Western blotting. We found that compared with the normoxic control, hypoxia increased COL10A1 and vimentin expression, but decreased miR-101, KLF4, and E-cadherin expression in HK-2 cells, while KLF4 overexpression decreased COL10A1 expression and mitigated the hypoxia-upregulated vimentin expression, but rescued miR-101 and E-cadherin expression in HK-2 cells (Figure 6C–F). Collectively, these data suggest that KLF4-induced pre-miR-101 expression mitigates the hypoxia-enhanced Col10A1 expression, EMT, fibrosis, and tubular cell injury, contributing to renal fibrosis after AKI.

**Table 1.** The primers for RT-qPCR.

Source	Primer	Direction	Sequence (5’-3’)
mmu	GAPDH	Forward	TGTTCCTACCCCCAATGTGTCCGTC
		Reverse	CTGGTCCTCAGTGTAGCCCAAGATG
	COL10A1	Forward	ATGCCCGTGTCTGCTTT
		Reverse	AGGCGTGCCGTTCTTAT
	Vimentin	Forward	CCCAGATTCAGGAACAGCATG
		Reverse	CCTGTCTCCGGTACTCGTTTGA
	E-cadherin	Forward	TCAAAGTGGCGACAGACGG
		Reverse	GTTGGATTCAGAGGCAGGGT
	KLF4	Forward	GGCGAGTCTGACATGGCTG
		Reverse	GCTGGACGCAGTGTCTTCTC
hsa	GAPDH	Forward	GACCTGACCTGCCGTCTAG
		Reverse	AGGAGTGGGTGTCGCTGT
	α-SMA	Forward	AGTTACGAGTTGCCTGATGG
		Reverse	TGATGCTGTTGTAGGTGGTT
	E-cadherin	Forward	GAACGCATTGCCACATACAC
		Reverse	TGGTGTAAGCGATGGCGGCA
	vimentin	Forward	TTGAACGCAAAGTGGAATC
		Reverse	AGGTCAGGCTTGGAAACA
	COL10A1	Forward	ATAGGAACTCCCATACCATTT
		Reverse	CACTCCCTGAAGCCTGAT
	KLF4	Forward	CCCACATGAAGCGACTTCCC
		Reverse	CAGGTCCAGGAGATCGTTGAA

## Discussion

Some new signaling pathways have been found to participate in the process of kidney injury. The Wnt/β-catenin signaling appears to be essential for minimizing initial kidney damages by promoting adaptive repair and regeneration after AKI [16]. Conversely, the TGF-β1/Smad signaling contributes to kidney damage by replacing nephron parenchyma with nonfunctional fibrotic tissues [17]. Interestingly, the Wnt and Notch expression prohibits epithelial differentiation whereas increased levels of Wnt and Hh expression induce fibroblast proliferation and myofibroblastic transdifferentiation [18]. FGFR1 is crucial for the LPS-induced inflammation in renal tubular epithelial cells [19]. Activation of SIRT3 attenuates the sepsis-induced AKI by modulating the AMPK/mTOR signaling to enhance autophagy [20]. However, the molecular mechanisms underlying the progression of AKI to renal fibrosis remain to be clarified.

COL10A1 is one of the collagens and is highly expressed in various types of cancers. Our previous study found that miR-101 suppresses AKI-CKD transition by inhibiting the process of EMT, accompanied by reduced COL10A1 and other fibrotic factor expression in mice [11]. Therefore, the miR-101/COL10A1 axis contributes to the pathogenesis of renal fibrosis in mice. Alterations in microRNA expression influence the EMT and EndMT programs by regulating the biological pathways and signaling events and maintaining the homeostasis: such as miR-199a-5p, miR-205, miR-504, miR-30c, miR-200 and miR-34 [21–24]. Interactions among miRNAs and other non-coding miRNAs and antifibrotic miRNAs also regulate the process of EMT and EndMT, for example, LincK regulates ZEB1 by sponging miR-200. Long stress-induced non-coding transcript 5 promotes cell motility through the Wnt signaling, by sponging the miR-30a-induced cellular proliferation, motility and EMT [25]; exosomes containing miR-29b mimic were effectively internalized by cardiac fibroblasts, enhancing the expression of miR-29b to prevent fibrosis progression and suppress the production of ECM proteins [26,27].

Inhibition of EMT effectively alleviates fibrosis in the kidneys [28]. COL10A1 is considered a non-fibrous collagen and a biomarker for several types of tumors [12,13]. In this study, we detected significantly upregulated COL10A1 expression in the kidney tissues of I/R mice, indicating that upregulation of COL10A1 expression enhanced renal fibrosis. Furthermore, we found that hypoxia increased COL10A1 and NGAL expression in HK-2 cells, further supporting the notion that COL10A1 contributes to renal fibrosis after AKI. More importantly, COL10A1 silencing significantly mitigated the hypoxia-upregulated NGAL expression in HK-2 cells. In addition, hypoxia decreased E-cadherin, but increased vimentin and α-SMA expression, promoting the EMT process in renal epithelial cells with a microfibroblast phenotype, consistent with a previous report [29]. In contrast, COL10A1 silencing significantly mitigated or abrogated the effects of hypoxia by enhancing E-cadherin expression, but reducing vimentin and α-SMA expression to inhibit the hypoxia-enhanced EMT process in HK-2 cells and preserve their epithelial cell phenotype. More importantly, COL10A1 silencing not only reduced morphological renal injury and interstitial fibrosis, Vimentin and NGAL expression, but also increased E-cadherin expression in the kidney tissues of I/R mice. Previous studies have shown that hyaluronic acid can inhibit the TGF-β-induced synovial fibrosis in a mouse model of arthritis by downregulating COL10A1 expression [30] and treatment with mignonette element-7 or glucuronic acid glycosides significantly reduces myocardial injury and fibrosis in a mouse model of myocardial fibrosis induced by isopropyl adrenaline by downregulating COL10A1 and fibrogenic factor gene expression [31]. Thus, COL10A1 may be a critical factor in the I/R-induced EMT and renal injury, at least in mice.

KLF4, a transcription factor, expression is downregulated during kidney injury [32], and KLF4 and some miRNAs have been reported to have renoprotective activities in rodent models of renal injury [33,34]. Actually, KLF4 silencing in macrophages deteriorates the renal tubulointerstitial inflammation, fibrosis and injury in mice [35,36]. KLF4 acts as a negative regulator of EMT and fibrosis in the kidneys. KLF4 over-expression enhanced pre-miR-101 promoter-controlled luciferase expression *in vitro*, consistent with bioinformatic prediction. Furthermore, KLF4 overexpression reduced COL10A1 expression in hypoxic HK-2 cells, and mitigated the hypoxia-enhanced vimentin expression and rescued E-cadherin expression. These data indicated that during AKI-related renal fibrosis, downregulation of KLF4 expression minimized miR-101 transcripts, in turn, to enhance COL10A1 expression, promoting the EMT process, renal fibrosis, and injury. Conceivably, the upregulation of KLF4 and miR-101 expression may be valuable for controlling EMT, attenuating fibrosis, and renal injury.

In summary, our data indicated that serum Scr, BUN, and NGAL levels, Col10a1 expression, and fibrosis increased in the kidney tissues of I/R mice, accompanied by enhanced EMT. Similarly, hypoxia induced the EMT process in renal epithelial cells, upregulated the expression of NGAL and COL10A1 in HK-2 cells. Hypoxia-induced EMT was significantly mitigated or abrogated by COL10A1 silencing in mice and HK-2 cells. Furthermore, KLF4 induced miR-101 expression to inhibit COL10A1 expression in renal tubular epithelial cells. While hypoxia downregulated KLF4 expression and enhanced the EMT process, KLF4 overexpression significantly mitigated or abrogated hypoxic effects in HK-2 cells. These novel findings unveil that KLF4 modulates the miR-101/COL10A1 axis to inhibit EMT and renal fibrosis after AKI. Therefore, our findings may shed light on the pathogenic process of renal fibrosis after AKI and uncover therapeutic targets for the development of new therapies for the treatment of CKD.

## Data Availability

The data supporting the findings of this study are available upon request from the corresponding author.

## References

[1] Coca SG, Singanamala S, Parikh CR. Chronic kidney disease after acute kidney injury: a systematic review and meta-analysis. Kidney Int. 2012;81(5):1–10. doi:10.1038/ki.2011.379. PMC3788581 22113526

[2] Tampe B, Steinle U, Tampe D, et al. Low-dose hydralazine prevents fibrosis in a murine model of acute kidney injury-to-chronic kidney disease progression. Kidney Int. 2017;91(1):157–176. doi:10.1016/j.kint.2016.07.042. 27692563

[3] Arai S, Kitada K, Yamazaki T, et al. Apoptosis inhibitor of macrophage protein enhances intraluminal debris clearance and ameliorates acute kidney injury in mice. Nat Med. 2016;22(2):183–193. doi:10.1038/nm.4012. 26726878

[4] Wang Z, Zhang W. Role of fatty acid oxidation in the pathogenesis and prognosis of acute kidney injury induced by ischemia reperfusion. Chinese J Nephrol. 2019;35(10):784–789.

[5] Liu BC, Tang TT, Lv LL, et al. Renal tubule injury: a driving force toward chronic kidney disease. Kidney Int. 2018;93(3):568–579. doi:10.1016/j.kint.2017.09.033. 29361307

[6] Venkatachalam MA, Weinberg JM, Kriz W, et al. Failed tubule recovery, AKI-CKD transition, and kidney disease progression. J Am Soc Nephrol. 2015;26(8):1765–1776. doi:10.1681/ASN.2015010006. 25810494 PMC4520181

[7] Iwakura T, Zhao Z, Marschner JA, et al. Dipeptidyl peptidase-4 inhibitor teneligliptin accelerates recovery from cisplatin-induced acute kidney injury by attenuating inflammation and promoting tubular regeneration. Nephrol Dial Transplant. 2019;34(10):1669–1680. doi:10.1093/ndt/gfy397. 30624740

[8] Li M, Li C-M, Ye Z-C, et al. Sirt3 modulates fatty acid oxidation and attenuates cisplatin-induced AKI in mice. J Cell Mol Med. 2020;24(9):5109–5121. doi:10.1111/jcmm.15148. 32281286 PMC7205836

[9] Shen Y, Jiang L, Wen P, et al. Tubule-derived lactate is required for fibroblast activation in acute kidney injury. Am J Physiol Renal Physiol. 2020;318(3):F689–F701. doi:10.1152/ajprenal.00229.2019. 31928224

[10] Barrera-Chimal J, André-Grégoire G, Nguyen Dinh Cat A, et al. Benefit of mineralocorticoid receptor antagonism in AKI: role of vascular smooth muscle Rac1. J Am Soc Nephrol. 2017;28(4):1216–1226. doi:10.1681/ASN.2016040477. 28087726 PMC5373452

[11] Zhao JY, Wang XL, Yang YC, et al. Upregulated miR-101 inhibits acute kidney injury-chronic kidney disease transition by regulating epithelial-mesenchymal transition. Hum Exp Toxicol. 2020;39(12):1628–1638. doi:10.1177/0960327120937334. 32633566

[12] Huang H, Li T, Ye G, et al. High expression of COL10A1 is associated with poor prognosis in colorectal cancer. Onco Targets Ther. 2018;11:1571–1581. doi:10.2147/OTT.S160196. 29593423 PMC5865565

[13] Li T, Huang H, Shi G, et al. TGF-β1-SOX9 axis-inducible COL10A1 promotes invasion and metastasis in gastric cancer via epithelial-to-mesenchymal transition. Cell Death Dis. 2018;9(9):849. doi:10.1038/s41419-018-0877-2. 30154451 PMC6113209

[14] Zhou W, Chen YX, Ke B, et al. circPlekha7 suppresses renal fibrosis via targeting miR-493-3p/KLF4. Epigenomics. 2022;14(4):199–217. doi:10.2217/epi-2021-0370. 35172608

[15] Zhao JY, Wu YB. Huaier extract attenuates acute kidney injury to chronic kidney disease transition by inhibiting endoplasmic reticulum stress and apoptosis via miR-1271 upregulation. Biomed Res Int. 2020;2020:9029868–9029868. doi:10.1155/2020/9029868. 33457422 PMC7787756

[16] Botros SR, Matouk AI, Anter A, et al. Protective effect of empagliflozin on gentamicin-induced acute renal injury via regulation of SIRT1/NF-κB signaling pathway. Environ Toxicol Pharmacol. 2022;94:103907. doi:10.1016/j.etap.2022.103907. 35697188

[17] Zhou D, Tan RJ, Fu H, et al. Wnt/β-catenin signaling in kidney injury and repair: a double-edged sword. Lab Invest. 2016;96(2):156–167. doi:10.1038/labinvest.2015.153. 26692289 PMC4731262

[18] Edeling M, Ragi G, Huang S, et al. Developmental signalling pathways in renal fibrosis: the roles of notch, wnt and hedgehog. Nat Rev Nephrol. 2016;12(7):426–439. doi:10.1038/nrneph.2016.54. 27140856 PMC5529143

[19] Chen X, Zhang X, Xu J, et al. AZD4547 attenuates lipopolysaccharide-induced acute kidney injury by inhibiting inflammation: the role of FGFR1 in renal tubular epithelial cells. Drug Des Devel Ther. 2020;14:833–844. doi:10.2147/DDDT.S224343. PMC7049771 32161443

[20] Zhao W, Zhang L, Chen R, et al. SIRT3 protects against acute kidney injury via AMPK/mTOR-regulated autophagy. Front Physiol. 2018;9:1526. doi:10.3389/fphys.2018.01526. 30487750 PMC6246697

[21] Pan G, Liu Y, Shang L, et al. EMT-associated microRNAs and their roles in cancer stemness and drug resistance. Cancer Commun. 2021;41(3):199–217. doi:10.1002/cac2.12138. PMC7968884 33506604

[22] Feng J, Hu S, Liu K, et al. The role of MicroRNA in the regulation of tumor epithelial–mesenchymal transition. Cells. 2022;11(13):1981. doi:10.3390/cells11131981. 35805066 PMC9265548

[23] Uhan S, Hauptman N. Metastatic EMT phenotype is governed by microRNA-200-mediated competing endogenous RNA networks. Cells. 2021;11(1):73. doi:10.3390/cells11010073. 35011635 PMC8749983

[24] Srivastava SP, Hedayat AF, Kanasaki K, et al. microRNA crosstalk influences epithelial-to-mesenchymal, endothelial-to-mesenchymal, and macrophage-to-mesenchymal transitions in the kidney. Front Pharmacol. 2019;10:904. doi:10.3389/fphar.2019.00904. 31474862 PMC6707424

[25] Venkatesh J, Wasson M-CD, Brown JM, et al. LncRNA-miRNA axes in breast cancer: novel points of interaction for strategic attack. Cancer Lett. 2021;509:81–88. doi:10.1016/j.canlet.2021.04.002. 33848519

[26] Yuan J, Yang H, Liu C, et al. Microneedle patch loaded with exosomes containing microRNA‐29b prevents cardiac fibrosis after myocardial infarction. Adv Healthc Mater. 2023;12(13):e2202959. doi:10.1002/adhm.202202959. 36739582

[27] Yan L, Su Y, Hsia I, et al. Delivery of anti-microRNA-21 by lung-targeted liposomes for pulmonary fibrosis treatment. Mol Ther Nucleic Acids. 2023;32:36–47. doi:10.1016/j.omtn.2023.02.031. 36919116 PMC9972768

[28] Chang JW, Tsai HL, Chen CW, et al. Conditioned mesenchymal stem cells attenuate progression of chronic kidney disease through inhibition of epithelial-to-mesenchymal transition and immune modulation. J Cell Mol Med. 2012;16(12):2935–2949. doi:10.1111/j.1582-4934.2012.01610.x. 22862802 PMC4393722

[29] Fan JM, Ng YY, Hill PA, et al. Transforming growth factor-beta regulates tubular epithelial-myofibroblast transdifferentiation in vitro. Kidney Int. 1999;56(4):1455–1467. doi:10.1046/j.1523-1755.1999.00656.x. 10504497

[30] Li J, Gorski DJ, Anemaet W, et al. Hyaluronan injection in murine osteoarthritis prevents TGFbeta 1-induced synovial neovascularization and fibrosis and maintains articular cartilage integrity by a CD44-dependent mechanism. Arthritis Res Ther. 2012;14(3):R151. doi:10.1186/ar3887. 22721434 PMC3446537

[31] Castro NE, Kato M, Park JT, et al. Transforming growth factor β1 (TGF-β1) enhances expression of profibrotic genes through a novel signaling Cascade and microRNAs in renal mesangial cells. J Biol Chem. 2014;289(42):29001–29013. doi:10.1074/jbc.M114.600783. 25204661 PMC4200256

[32] Bertero A, Brown S, Madrigal P, et al. The SMAD2/3 interactome reveals that TGFβ controls m(6)a mRNA methylation in pluripotency. Nature. 2018;555(7695):256–259. doi:10.1038/nature25784. 29489750 PMC5951268

[33] Yang X, Li B, Guan Y, et al. Expressions and related mechanisms of miR-212 and KLF4 in rats with acute kidney injury. Mol Cell Biochem. 2021;476(4):1741–1749. doi:10.1007/s11010-020-04016-x. 33428060

[34] Xu D, Chen P-P, Zheng P-Q, et al. KLF4 initiates sustained Yap activation to promote renal fibrosis in mice after ischemia-reperfusion kidney injury. Acta Pharmacol Sin. 2021;42(3):436–450. doi:10.1038/s41401-020-0463-x. 32647339 PMC8027004

[35] Rane MJ, Zhao Y, Cai L. Krϋppel-like factors (KLFs) in renal physiology and disease. EBioMedicine. 2019;40:743–750. doi:10.1016/j.ebiom.2019.01.021. 30662001 PMC6414320

[36] Wen Y, Lu X, Ren J, et al. KLF4 in macrophages attenuates TNFα-mediated kidney injury and fibrosis. J Am Soc Nephrol. 2019;30(10):1925–1938. doi:10.1681/ASN.2019020111. 31337692 PMC6779357

